# Comparative Performance Analysis of Support Vector Machine, Random Forest, Logistic Regression and *k*-Nearest Neighbours in Rainbow Trout (*Oncorhynchus Mykiss*) Classification Using Image-Based Features

**DOI:** 10.3390/s18041027

**Published:** 2018-03-29

**Authors:** Mohammadmehdi Saberioon, Petr Císař, Laurent Labbé, Pavel Souček, Pablo Pelissier, Thierry Kerneis

**Affiliations:** 1 Institute of Complex Systems, South Bohemian Research Centre of Aquaculture and Biodiversity of Hydrocenoses, Faculty of Fisheries and Protection of Waters, University of South Bohemia in České Budějovice, Zámek 136, Nové Hrady 37 333, Czech Republic; cisar@frov.jcu.cz (P.C.); psoucek@frov.jcu.cz (P.S.); 2 Institut National de la Recherche Agronomique (INRA), UE 0937 PEIMA (Pisciculture Expérimentale INRA des Monts d’Arrée), 29450 Sizun, France; Laurent.labbe@inra.fr (L.L.); pablo.pelissier@inra.fr (P.P.); thierry.kerneis@inra.fr (T.K.)

**Keywords:** image colour properties, image texture properties, machine vision system, supervised classification, image processing

## Abstract

The main aim of this study was to develop a new objective method for evaluating the impacts of different diets on the live fish skin using image-based features. In total, one-hundred and sixty rainbow trout (*Oncorhynchus mykiss*) were fed either a fish-meal based diet (80 fish) or a 100% plant-based diet (80 fish) and photographed using consumer-grade digital camera. Twenty-three colour features and four texture features were extracted. Four different classification methods were used to evaluate fish diets including Random forest (RF), Support vector machine (SVM), Logistic regression (LR) and *k*-Nearest neighbours (*k*-NN). The SVM with radial based kernel provided the best classifier with correct classification rate (CCR) of 82% and Kappa coefficient of 0.65. Although the both LR and RF methods were less accurate than SVM, they achieved good classification with CCR 75% and 70% respectively. The *k*-NN was the least accurate (40%) classification model. Overall, it can be concluded that consumer-grade digital cameras could be employed as the fast, accurate and non-invasive sensor for classifying rainbow trout based on their diets. Furthermore, these was a close association between image-based features and fish diet received during cultivation. These procedures can be used as non-invasive, accurate and precise approaches for monitoring fish status during the cultivation by evaluating diet’s effects on fish skin.

## 1. Introduction

Food quality and methods of production have become primary concerns relative to consumer behaviour and increased industrialization and globalization of the food supply chain. Consumers demand high quality and safety in fish and fish products which requires high standards in process control and quality assurance [1]. 

Criteria for quality of fish and fish products can be divided into external and internal traits. External traits can be measured by estimating body mass, slaughter yield and proportion of fillet and carcass. Internal traits are assessed by evaluating chemical properties, such as the water contents and total amount of lipids and proteins and physical properties such as cooking loss, relative shear force, flesh lightness and colour [2]. Colour is more important because it is closely related to consumer perception for evaluating the freshness, quality and better flavour in fish and fish products [3]. For instance, consumers prefer rainbow trout (*Oncorhynchus mykiss*) with blue-black phenotype in some market, due to their higher growth rates (+23%) in comparison to other skin colour phenotypes [4]. Yi et al. [5] reported that one of the main reasons large yellow croaker (*Larimichthys croceus* R) has a lower market price and consumer acceptance is that it loses its natural colour when intensively cultivated. Several factors can affect fish skin colour, namely ingredients in the feed [5,6], environmental colour [7] and pre-harvest processing [8].

Currently, several direct and indirect methods and instruments are available for assessing colour of treated fish and aquatic products such as sensory panels, colorimeters and machine vision systems. Sensory panels can be used to evaluate the colour. It is a simple, inexpensive, quick and non-destructive method for quantifying the changes in fish skin; however, it is labour intensive, inaccurate and is difficult to quantify [9]. Colorimeters been used to measure skin colour; these instruments usually provide readings in XYZ, RGB and CIE Lab colour space, allow accurate and reproducible measurements of the colour with no influence by the observed or surroundings [10]. For instance, Skonberg et al. [11] used a colorimeter to analysis and discriminate fillets of fish which received either wheat gluten or corn gluten and Macagnano et al. [12] evaluated freshness based on fish skin colour. Kalinowski et al. [13] used a tristimulus colorimeter to characterize the intensity of skin colour parameters (CIE Lab) to determine the effect of esterified astaxanthin supplement in red coloration of red porgy. Yi et al. [5] used portable Minolta Chroma meter to evaluate the effects of astaxanthin and xanthophylls as carotenoid sources on growth and skin colour of large yellow croaker. Although fish skin colour described with colorimeters is accurate, a relatively small area is measured by the machine and thus some aspects of the overall colours are lost [14]. Also, for complete measurement, many locations on the sample must be measured to obtain the representative colour profile or the surface colour should be quite uniform or homogenous [15]. 

During the past decade, machine vision system (MVS) have been used exclusively for quality assessment of fish and fish products. MVS can extract and analyses quantitate information from digital images. MVS is a comprehensive technology which consists of two main components namely image acquisition system and image processing. These are considered not only to be at least good as colorimeters but also can overcome the deficiencies of colorimeters for evaluating fish and fish products parameters [16]. Zaťková et al. [17] showed the feasibility of a machine vision system for monitoring skin colour changes due to diet alteration in ornamental fish species. Colihueque [18] presented the capability of a machine vision system for estimating skin colour and spottiness of rainbow trout for categorizing at juvenile stages. Balaban et al. [19] also used a machine vision system to quantify the skin colour changes of snapper (*Pagrus auratus*) and gurnard (*Chelidonichthys kumu*) due to cold storage. Segade et al. [6] showed the effects of different diets on seahorse (*Hippocampus hippocampus*) body colour using the machine vision system. Wishkerman et al. [20] used the machine vision system to extract and classify the albinism based on skin colour and texture features in fish. Wallat et al. [21] also employed a machine vision system that was designed by Luzuriaga et al. [22] to measure skin colour of gold fish (*Carassius auratus*) for optimizing diets to achieve the most desirable skin colour. 

The most essential component of machine vision system is machine learning (ML) algorithms. It provides a mechanism in which the human thinking process is simulated artificially and can assist in human decision making more accurately, quickly and consistently [23]. ML algorithms are usually employed to either trivial or nontrivial relationships in a set of training data automatically, which produces the generalization of these relationships that can be used to interpret new datasets. Currently several studies use different machine learning algorithms to classify or discriminate aquatic animals based on different image features such as colour and geometrical or morphological features in aquaculture and marine science. For example, Hu et al. [24] employed multi-class support vector machine (SVM) to classify six different fresh water fish species based on their skin colour and texture. They showed that multi-class SVM could classify fish species with high accuracy rate (97.77%). Hernández-Serna and Jiménez-Segura, [25] developed an automated identification system to classify fish species with 91.86% accuracy. They utilized geometrical, morphological and textural characteristics of images, together with artificial neural network (ANN) as machine learning algorithm for classification. Rossi et al. [26] also developed another system for the identification of fish species called FishAPP using a machine vision system coupled with ANN. Liu et al. [27] used a machine vision system and improved the majority rule (IMAJ) classifier to identify impurities in fresh shrimp. They showed that IMAJ based classifier combined with parallel features are superior (91.53%) over other fusion rule-based classifiers. Dutta et al. [28] used support vector machine and machine vision system to identify pesticide residues in fish with high accuracy (95%). Wishkerman et al. [20] used combination of Gray Level Co-Occurrence Matrix (GLOM) and data reduction procedures such as principal component analysis (PCA) and linear discriminant analysis (LDA) to discriminate pseudo-albinosis in Senegalese sole. 

The main objective of this study was to evaluate the feasibility of machine vision system to predict the fish diet based on their skin image as inexpensive, non-invasive and rapid approach. To the best of our knowledge, no studies have been done on evaluating the impacts of different diets on the live fish skin using image-based features. This method will be of great value for evaluating fish nutrition and fish welfare studies. Another objective of this study was to compare the performance of different machine learning algorithms for classifying rainbow trout based on their diets to find the most accurate image processing methods.

## 2. Materials and Methods

### 2.1. Fish and Cultural Condition

The experimental groups were produced at INRA-PEIMA (Sizun, France). It contained 160 fish which were grown in six 1.8 m^3^ replicated tanks supplied by river water (13.4–18.3 °C) until data acquisition. All fish were tagged with passive integrated transponder (AEG-Id, ISO FDXB) for individual identification. Experimental design was a split-block design with three replications for each diet; therefore, 80 fish were fed a fish-meal based diet (3 tanks) and 80 were fed a plant-based diet (3 tanks). After three weeks, all fish from each treatment were used for image acquisition. Mean body weight of fish receiving the fish-meal based diet (FBD) was 228.99 g and the plant-based diet (PBD) was 222.46 g at time of data acquisition. Experiment has been approved by French veterinary service under ethical approval number B-29-277-02. The experiment was in strict accordance with EU legal framework related to the protection of animals used for scientific research (Directive 2010/63/EU) and according to the National guidance for the animal care of the French ministry of research (Decree no. 2001-464, 29 May 2001).

### 2.2. Diets and Feeding Controls

Diets were manufactured at the INRA NUMEA facility of Donzacq (Paris, France). The ingredient and analysis composition are given in Table 1. FBD contained fishmeal and fish oil as protein and lipid source respectively. PBD contained a mixture of wheat gluten, extruded peas, corn gluten meal, soybean meal and white lupin as protein sources; and a combination of palm seed, rapeseed and linseed oil, rich in saturated, mono-unsaturated and n-3 poly-unsaturated fatty acids, as lipid source. A mineral and a vitamin premix equally were added into both diets. Both diets fulfilled the known nutrient requirement of rainbow trout as explained by National Research Council, [29].

### 2.3. Image Acquisition

Before measurement, each fish was mildly anesthetized with Benzocaine to reduce the movement and minimize stress. The surface of each rainbow trout was wiped with a piece of tissue paper to remove extra water from the skin before data acquisition. Each live fish was photographed with a 12-megapixel Nikon D3300 digital camera (Nikon Corp., Tokyo, Japan) under a lighting system consisting of four halogen lamps (200 W bulb) at an angle of 45 degrees and 35 cm above the sample to not only provide constant intensity output but also to give the uniform light intensity over the fish sample. Images were collected in a dark room with only light source, coming through halogen lamps cast on fish skin. Digital camera was located vertically at 56 cm from the sample. All setting on the camera were on manual. The setting of camera was: exposure mode = manual, shutter speed = 1/160 s, aperture = f/4.0, ISO sensitivity = 100. The images were recorded in Nikon Raw format (NEF) and transferred to a laptop for further processing. To calibrate the colour of the image, colour checker (Gretag Color Checker, X-Rite Inc., Grand Rapids, MI, USA) was used as reference. The calibration provides a means of transforming the acquired images to a standard and well-defined colour spaces. In this study, CIEL*a*b* colorimetric space as well-defined colour space used to compare and compute of perceptual colour differences. 

### 2.4. Image-Based Feature Extraction

As Yang et al. [30] suggested a combination of multiple image-extracted features can enhance the performance of image processing systems, therefore, colour and texture features were extracted as image features to use to train classifiers.

#### 2.4.1. Colour Feature Extraction

**Figure 1:**
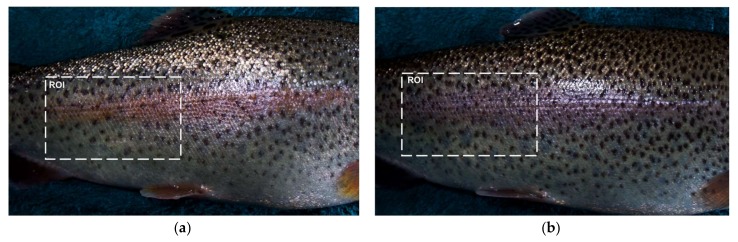
Sample image of rainbow trout and selected region of interest (ROI) (**a**) PBD (**b**) FBD.

Several studies showed that diet has an impact on fish skin colour [5,6], besides extensive research highlighted that colour spaces and indices were powerful features for fish classification [8,31,32], thus in the current study, 160 images were analysed to calculate twenty-three colour parameters. The region of interest (ROI) was selected manually from the whole image. In the selected ROI, tried to avoid background, the saturated pixels and the edge (Figure 1). The colour parameters were obtained from the average colour of whole ROI. The original image stored by camera were converted to the RGB colour space and other colour spaces and indices were calculated from this representation. RGB and HSV colour spaces were calculated using Matlab as employed by Tang et al. [33]. Meanwhile, the components of the CIELa*b* colour space were calculated according to the procedure by Trussell et al. [34]. CIE 1931 XYZ colour space also calculated as explained by Westland and Ripamonti [35]. To reduce the effect of illumination, the normalized RGB (rgb) values were also calculated as normalization [36]. Other known colour indices also calculated as covariant in this study. All colour spaces and colour indices used for classification are listed and defined in Table 2.

#### 2.4.2. Image Texture Extraction

As Haidekker, [41] and Wishkerman et al. [20] pointed out, texture analysis can be used for differentiating between two different condition from different images. Therefore, the Gray Level Co-occurrence Matrix (GLCM) was used to extract four second-order statistical texture features; 1/Contrast 2/Energy 3/Homogeneity 4/Correlation. All texture features and their description are listed in Table 3. Further details can be found in Hall-Beyer, [42].

### 2.5. Classifiers

Four different classifiers, Support vector machine (SVM), Random forest (RF), Logistic regression (LR) and *k*-Nearest Neighbour (*k*-NN) were applied to classify colour and texture features extracted from live fish skin so as to categorize fish based on their diet received during the test. All four models can be divided into two groups based on their interpretability namely: simple and complex. Simple models, such as *k*-NN and LR, have few parameters and are interpretable but on other hand, complex models such as SVM and RF are complex, difficult to interpret and have many parameters. The summary of each algorithm is presented in following sections.

#### 2.5.1. Support Vector Machine (SVM)

Support Vector Machine (SVM) is a nonparametric, supervised and kernel-based method from statistical learning methods. Kernel-based learning uses an implicit mapping of the input data into a high-dimensional feature-space described by a kernel function. In other words, kernel-based learning uses linear hyperplane as a decision function for nonlinear problems and then applies a back transformation in nonlinear space. SVM employs the Lagrange multiplier to compute the partial differentiation of each feature to acquire the optimal solution. In consequence, the model reduces the complexity of the training data to a significant subset of so-called support vectors. Consider a given training set of *N* data points, $\{x_{k},\;y_{k}{\}}_{k=1}^{N}$ with input data, which is an n-dimensional data vector (x_k∈R^N) and output, which is the one-dimensional vector space (y_k∈r); SVM create the classifier as shown in Equation (1).

(1) $$y\left(x\right)=sign\left[\overset{N}{\underset{k=1}{\sum }}\alpha_{k}y_{k}\psi \left(x,\;x_{k}\right)+b\right]$$

where $\alpha_{k}$ are positive real constants and *b* is a real constant. For this study, SVM with radial basis function was used as one of the popular kernel. Radial basis function can be calculated using Equation (2).

(2) $$\psi \left(x,\;x_{k}\right)=exp\left\{-\frac{\left\|{\left(x-x_{k}\right)}^{2}\right\|}{2\sigma^{2}}\right\},\;k=1,\ldots ,\;N$$

where *σ* is width of the radial basis function which were determined by a grid search method using repeated cross validation approach. Further details can be found in Hsu et al. [43] and Vapnik, [44]. R package Caret [45] used for SVM classification model.

#### 2.5.2. Random Forest (RF)

RF is a supervised and tree-based ensemble machine learning approach used in this study. RF is a theoretical framework grounded on mixture of decision trees; {*T*_1_(*X*), …, *T_B_*(*X*)}, where *X* = {*x*_1_, …, *x_p_*} is a *p*-dimensional vector of fish skin colour features, combining the concept of boosting or bootstrap aggregation (i.e., subsampling input samples with replacement) [46] and random subspace method (i.e., subsampling the variables without replacement) [47] applied at each split in the tree. The ensemble produces *B* outputs $\left\{{\overset{ˇ}{Y}}_{1}=T_{1}\left(X\right),\;\ldots ,\;{\overset{ˇ}{Y}}_{B}=T_{B}\left(X\right)\right\},\;\mathrm{where}\;{\overset{ˇ}{Y}}_{b},\;b=1,\ldots ,B$ is the prediction weight by the *b*th tree. Outputs of all trees are aggregated to produce one final prediction, $\overset{ˇ}{Y}$, which is the class predicted by majority of trees [48]. Similar to SVM, RF does not over-fit and it has robustness to noise and irrelevant features and almost no fine-tuning of parameters is needed to produce good predictions [49]. R package RandomForest [50] is used for prediction modelling.

#### 2.5.3. Logistic Regression (LR)

Generally, logistic regression (LR) calculate the class membership probability for two categories by fitting the log odds and explanatory variables to model using Equation (3)

(3) $$\log(\frac{P\left(Y=1|X\right)}{1-P\left(Y=1|X\right)})=\beta_{0}+\beta_{1}X_{1}+\ldots +\beta_{N}X_{N}$$

where *Y* = (0, 1) is the binary variable; 1 if it is higher than the Reference level and 0 if not, *X* = (*X*_1_, …, *X_n_*) are *n* explanatory variables which selected based on the Akaike Information Criterion (AIC) [51] and *β* = (*β*_0_, …, *β_n_*) are the estimated regression coefficient. Further details can be found in James et al. [52] R package glm2 [53] used for LR classification model.

#### 2.5.4. *k*-Nearest Neighbours (*k*-NN)

The *k*-Nearest Neighbours (*k*-NN) is another nonparametric method which predict the class of an object according to the class of its *k* nearest neighbours. *k*-NN is performed in three stages, firstly compute the distance (*N*_0_) from an observation *y_i_* to the all other observations *y_j_* using the distance function. In this study, Euclidean was used as distance function. It then estimates the conditional probability for class *j* as the fraction of points in *N*_0_ whose response values equal *j*:

(4) $$\mathrm{Pr}(Y=j|X=x_{0})=\frac{1}{k}\;\underset{i\in N_{0}}{\sum }I\left(y_{i}=j\right)$$

Afterward, the determination of class using those neighbours based on Bayes rule. Further details about *k*-NN can be found in James et al. [52]. R package Class [54] was used for *k*-NN implementation.

### 2.6. Evaluation of the Classification Models

Validation is an important component to test the learning status of the model. The dataset from 160 images for rainbow trout was divided into training set (80% of total samples) used to develop the classifier models and a validation set (20% of total samples) used to assess the prediction accuracy of each model. The training set was used for fitting models and the validation set was performed by random stratified sampling. Afterward, classifier was evaluated through the analysis of correct classification rate (CCR, %) and Cohen’s Kappa coefficient in the validation set. CCR and Cohen’s Kappa coefficient was calculated by the Equations (5) and (6) respectively.

(5) $$CCR=N_{1}/N_{0}\times 100\%$$

where *N*_1_ is number of corrected estimation of samples and *N*_0_ is the total number of samples.

(6) $$K=\frac{\mathrm{Pr}\left(a\right)-\mathrm{Pr}\left(e\right)}{1-\mathrm{Pr}\left(e\right)}$$

Furthermore, sensitivity and specificity which can be obtained using Equations (7) and (8) respectively; these were used to evaluate the classification model as well [55]. Sensitivity is the proportion of samples detected as positive that actually are positive, whereas the specificity is the proportion of negative samples that are correctly identified.

(7) Sensitivity=TP/(TP+FN)

(8) Specifity=TN/(FP+TN)

where *TP* and *TN* are true positive and true negative respectively; and *FN* and *FP* are false negative and false positive respectively.

**Figure 2:**
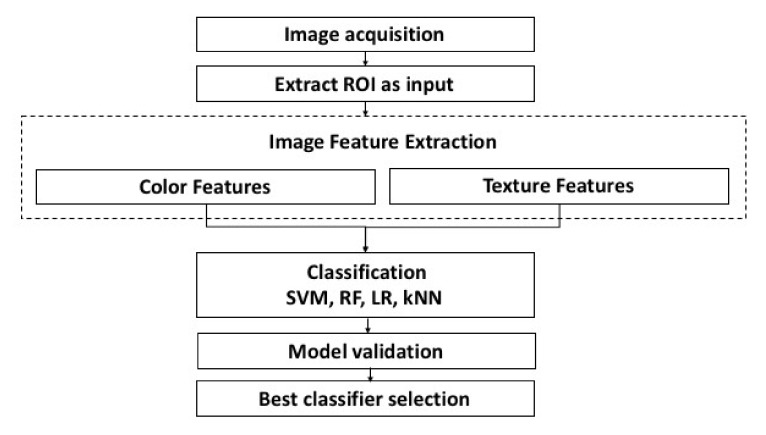
Flowchart of proposed fish classification methodology.

Additionally, the area under the Receiver Operator Characteristics (ROC) curve (AUC), known as a global measures of classifier performance, were calculated for comparing overall performance of all different classification schemes [56]. R package pROC [57] was used in this study to create ROC curves. Figure 2 shows the schematic of methodology used in this study.

## 3. Results

**Figure 3:**
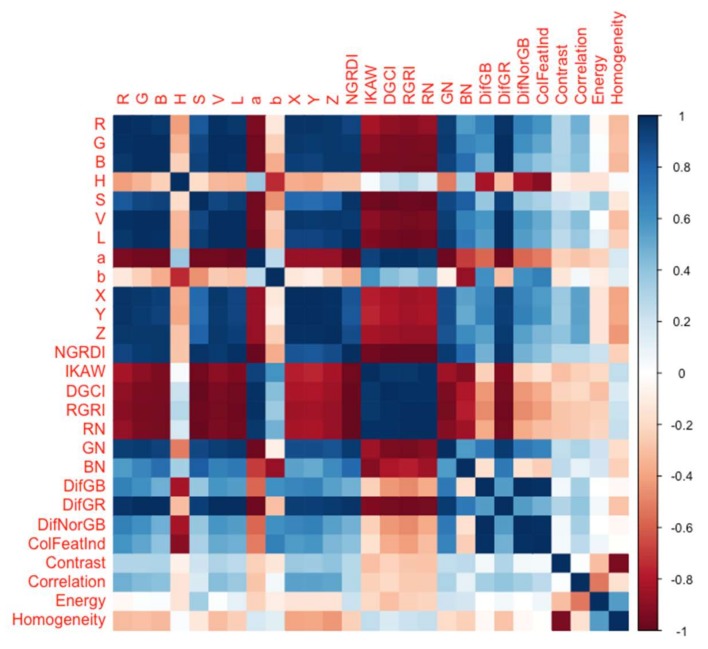
Correlation matrix of image-based features.

As mentioned previously, 23 colour features and 4 texture features (in total 27 image-based features) were extracted from each image. Matrix correlations were represented to obtain image-based features correlation to each other (Figure 3). Pearson’s two-tailed test for image-based features showed that colour features had significant correlation with each other however, they didn’t have significant correlation with texture features. Furthermore, significant negative correlation was seen between Homogeneity and Contrast but there was no significant correlation among other texture features.

Afterward, all extracted features were used for classification. The accuracy of classification models was evaluated by CCR, Kappa coefficient, Sensitivity and Specificity. Table 4 shows the average CCRs of the testing set for different classifiers. Range of CCR values for all machine learning algorithms are between 55% and 82%. SVM with Radial kernel demonstrated the best model with CCR of 82% and Kappa coefficient of 0.65 for testing set. In other words, results indicated that SVM had the highest probability to correctly classify fish to correct diet. Yet, Both LR and RF achieved good classification accuracy with CCR 75% and 70% respectively. *k*-NN displayed the overall lowest CCR (40%) and Kappa coefficient (0.2) which suggests that *k*-NN has no potential to discriminate between different groups of fish which received different diets. 

RF had the highest sensitivity, indicating that 70% of samples were detected as positive among those which were actually positive, whereas SVM and LR can correctly identified 65% samples as positive. The highest specificity is for SVM model explaining that 100% true negative samples were correctly identified. Overall high values of sensitivity and specificity acquired by classifiers except *k*-NN provide strong evidence that these models are robust and promising. 

**Figure 4:**
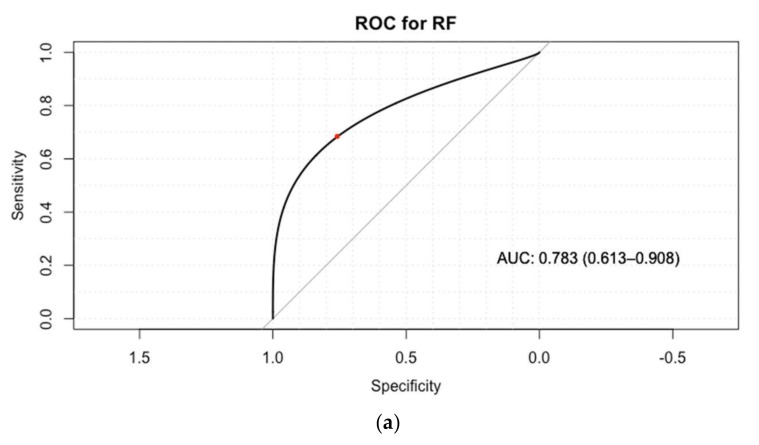

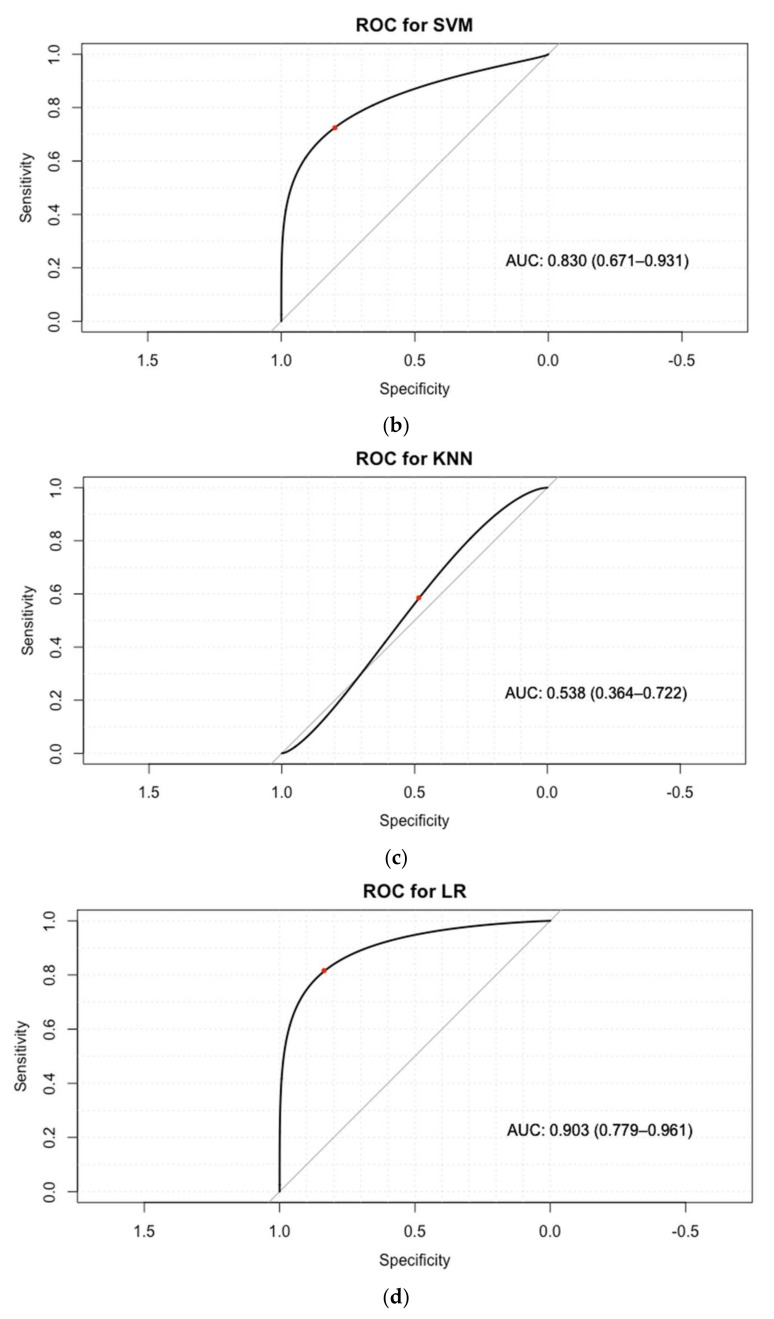
Receiver open characteristics (ROC) curves and measured area under curve (AUC) obtained for the test set of (**a**) Random forest; (**b**) Support vector machine; (**c**) *k*-Nearest Neighbour; (**d**) Logistic regression.

The ROC curves are also showed on Figure 4. ROC displays the variable overall performance of the classification as its discrimination threshold (relationship between specificity and sensitivity). The red dot on the figures is the closest point to the top corner where the true positive rate equals one and the false positive rate of zero [58]. Generally, the top corner point resulted from optimal threshold. Furthermore, AUC as the general quality index of classifiers mentioned. AUC of one is considered as a perfect classifier, while 0.5 would be a random classifier. Based on the AUC comparison of classifiers, performance of LR was 0.903 which was the highest and followed by SVM (0.830), RF (0.783) and *k*-NN (0.538) was the lowest. These results suggest that LR, SVM and RF have sufficient performance (AUC > 0.7), while *k*-NN had the weak performance.

**Figure 5:**
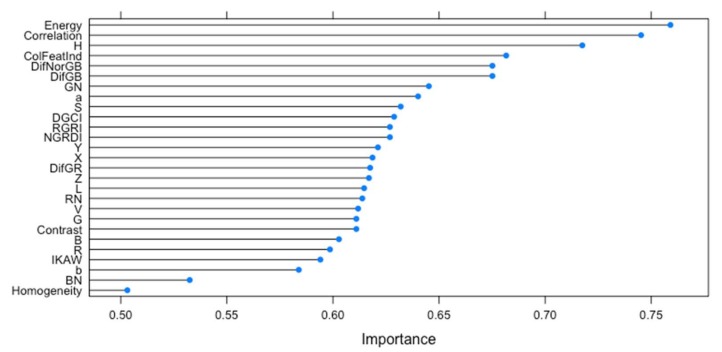
Rank of features by importance based on support vector machine (SVM) algorithm.

Additionally, AUC for each predictor computed and used as the measure of variable importance. The variable importance for all image-based features showed in Figure 5. It shows that Energy, Correlation and Hue (H) are the top 3 most important features in the dataset and Homogeneity is the least important image-based features. 

## 4. Discussion

The premier performance of SVM can be explained by its capability to minimize classification errors on unseen data without prior assumption made on the probability distribution the data. It also had the capability to derive a linear hyperplane as a decision function for nonlinear problems, which can be considered as another reason for selecting the method [59,60]. Furthermore, SVM-based classification can strike balance between acquired from a given finite amount of training patterns and the ability to generalize to unseen data [61]. 

The results showed that different diets had significant alteration to fish skin which were in line with similar studies [5,21,62,63,64]. More specifically, different dietary oil sources, which have different amounts of stanol and sterols might influence the absorption and deposition of carotenoids in fish skin [65,66,67]. 

This study also suggests that image features acquired by a consumer-grade digital camera and subsequent analysis by machine learning algorithms can be important tools for determining fish feed intake and fish welfare. Digital cameras provide a non-invasive, rapid and accurate method for classifying fish based on its diet. Quality of farmed fish are greatly influenced by management methods of culture and quality of farming environment [68] but also by the quality of feed and feed management [69], thus, utilizing introduced method might provide analytical approach to detect the source of feed for better discrimination of fish to have more accurate traceability system in aquaculture [70]. Furthermore, as Colihueque [18] pointed out, skin coloration and texture in farmed rainbow trout can be considered as one of the productive traits of commercial value because of their strong visual impact on the marketing prospects. Thus, the proposed methodology in this study can contribute to efforts to improve skin colour through diet manipulation.

## 5. Conclusions

This paper analysed and compared four popular machine learning approaches, including SVM, RF, LR and *k*-NN to evaluate fish based on their diet during culture using image-based features. The complex models were consistently better classifiers than simple models, thus complex models are recommended for characterizing fish based on their diets using their image-based features. This study revealed a close association between image-based features when coupled with SVM and the diet which fish received during cultivation. In other words, image-based features can be considered as reliable representative of fish skin for predicting diet.

The results of this study indicate that the consumer-grade digital camera could be employed for fast, accurate, inexpensive and non-invasive sensor for monitoring feed intake by quantifying external appearance of fish during the different growth stages. Furthermore, it introduces a method for better operation of traceability systems in aquaculture by providing cheaper and faster technique for discriminating different fish based on their source of diet. Furthermore, results of this study will pave the way for implementing precision fish farming concepts [71] by providing objective, fast, non-invasive and accurate approach for quantifying feed intake by fish during cultivation which would lead to feed optimization, better waste management and ultimately more sustainable aquaculture. Finally, this study can be extended further to investigate impacts of other types of diets on fish skin but also to assess the effect of different amount of one diet on fish skin. Moreover, additional studies should be conducted on different image processing method such as data reduction methods (e.g., PCA or LDA) or feature selection approaches (e.g., Rough-set-based algorithm [72]) to improve accuracy.

## Figures and Tables

**Table 1:** The Ingredient of fish meal-based diet (FBD) and plant-based diet (PBD).

Ingredients	FBD	PBD
Fish Oil	11.8	-
Plant oil blend ^1^	-	11.4
Fish meal	42.4	-
Soybean Meal	12.0	12.0
Pea	17.1	12.5
Wheat	9.6	4.0
Lupin flour	-	5.0
Wheat gluten	-	17.0
Corn gluten	-	17.0
Faba bean protein concentration	-	10.0
Dicalcium Phosphate	-	3.0
Soy lecithin powder	-	2.0
Additive (vitamin, mineral, preservative)	4.5	4.5

^1^ Palm seed, rapeseed and liveseed oil.

**Table 2:** Colour spaces and colour indices.

Name	Abbreviation	Definition	References
Red	R	Non-normalized Red	
Green	G	Non-normalized Green	
Blue	B	Non-normalized Blue	
Hue	H	Hue = W if B ≤ G or Hue 2 pi − W if B > G	[33]
Saturation	S	SAT = 1 − 3 min {r, g, b}	[33]
Value	V		[33]
Lightness	L		[34]
a *	a		[34]
b *	b		[34]
X	X		
Y	Y		
Z	Z		
Normalized Red	r	r = R*/(R + G + B) R* = Normalized R value (0–1), defined as R* = R/Rm (Rm = 255)	[37]
Normalized Blue	b	g = G*/(R + G + B) G* = Normalized G value (0–1), defined as G* = G/Gm (Gm = 255)	[37]
Normalized Green	g	b = B*/(R + G + B) B* = Normalized B value (0–1), defined as B* = B/Bm (Bm = 255)	[37]
Normalized green red difference index	NGRDI	NGRDI = (g − r)/(g + r)	[38]
Kawashima index	I_KAW_	I_kaw_ = R − B/R + B	[39]
Dark green colour index	DGCI	DGCI = {(Hue − 60)/60 + (1 − saturation) + (1 − brightness)/3	[40]
Red green ratio index	RGRI	RGRI = R/G	[37]
Difference between green and blue		G-B	[37]
Difference between Green and red		G-R	[37]
Difference between normalized green and normalized blue		g-b	
Colour feature index	G/B	G/B	[37]

**Table 3:** Texture features.

Feature	Description	Equation
Contrast	Shows Intensity contrast between a pixel and its neighbour over the whole image. Constant image has 0 value	$\underset{i,j}{\sum }{\left|i-j\right|}^{2}p\left(i,j\right)$
Energy	Shows sum of squared elements in the GLCM; it has range between 0 and 1 and 1 means constant image	$\underset{i,j}{\sum }p{\left(i,j\right)}^{2}$
Homogeneity	The closeness of the distribution of elements in the GLCM to the GLCM diagonal. It has range between 0 and 1 and GLCM diagonal has 1 as value.	$\underset{i,j}{\sum }\frac{p\left(i,j\right)}{1+\left|i-j\right|}$
Correlation	Shows correlation a pixel to its neighbours over the whole image. NaN is for constant image.	$\underset{i,j}{\sum }\frac{\left(i-\mu i\right)\left(j-\mu j\right)p\left(i,j\right)}{\sigma_{i}\sigma_{j}}$

**Table 4:** Model performance for identification of different diet on validation set.

Classifier	CCR%	Kappa	Sensitivity	Specificity
RF	70	0.40	0.70	0.70
SVM	82	0.65	0.65	1
*k*-NN	40	0.2	0.45	0.35
LR	75	0.50	0.65	0.85

## Supplementary Materials

All images acquired in this experiment are available online at https://doi.org/10.6084/m9.figshare.5978392.v1.
